# Oxidative dyslipidemia in early pregnancy: integrating atherogenic index of plasma (AIP) and uric acid (UA) to refine preeclampsia risk stratification

**DOI:** 10.3389/fphar.2026.1792375

**Published:** 2026-05-04

**Authors:** Nahui Samanta Nájera-Segura, María Teresa Hernández-Huerta, Eduardo Pérez-Campos, Efrén Emmanuel Jarquín González, Adrián Martínez-Vargas, Laura Pérez-Campos Mayoral, Wendy Reyna González, Ulises Jesús Roldán Trejo, Serafina Pérez Rodríguez, Heerajnarain Bulluck, Héctor Martínez Ruíz, Hector Alejandro Cabrera-Fuentes

**Affiliations:** 1 Centro de Investigación Facultad de Medicina UNAM-UABJO, Facultad de Medicina y Cirugía, Universidad Autónoma “Benito Juárez” de Oaxaca, Oaxaca, Mexico; 2 División de Estudios de Posgrado e Investigación, Tecnológico Nacional de México, Instituto Tecnológico del Valle de Etla, Oaxaca, Mexico; 3 SECIHTI, Faculty of Medicine and Surgery, Autonomous University “Benito Juárez” of Oaxaca, Oaxaca, Mexico; 4 División de Estudios de Posgrado e Investigación, Tecnológico Nacional de México, Instituto Tecnológico de Oaxaca, Oaxaca de Juárez, Oaxaca, Mexico; 5 Dirección General de los Servicios de Salud de Oaxaca, Secretaria de Salud, Servicios de Salud de Oaxaca, Oaxaca, Mexico; 6 Yorkshire Heart Centre, Leeds General Infirmary, Leeds Teaching Hospitals NHS Trust, Leeds, United Kingdom; 7 Leeds Institute of Cardiovascular and Metabolic Medicine, University of Leeds, Leeds, United Kingdom; 8 Hospital General de Zona No.1 Dr. Demetrio Mayoral Pardo, Instituto Mexicano del Seguro Social (IMSS), Oaxaca, Mexico; 9 R&D group, Vice Presidency Scientific Research & Innovation, Imam Abdulrahman bin Faisal University (IAU), Dammam, Saudi Arabia

**Keywords:** atherogenic index of plasma, biomarkers, first-trimester screening, maternal vascular dysfunction, metabolic risk stratification, oxidative dyslipidemia, preeclampsia, uric acid

## Abstract

Preeclampsia represents an unmasking of underlying vascular and metabolic vulnerability during the physiologic stress of pregnancy. Current first-trimester screening relies heavily on body mass index which incompletely captures qualitative metabolic dysfunction, particularly in non-obese women. In this narrative perspective, we examine the atherogenic index of plasma (AIP; log_10_[TG/HDL-C]), which reflects triglyceride-rich lipoprotein burden and has emerged as a marker of lipid-driven vascular stress independent of adiposity. This article does not follow systematic review methodology (e.g., PRISMA) and is intended to provide a conceptual synthesis rather than an exhaustive review. Emerging obstetric evidence suggests that first-trimester AIP (≤14 weeks) is associated with an increased risk of subsequent preeclampsia, with uric acid (UA) potentially amplifying this relationship through redox-dependent mechanisms. We propose that elevated AIP and UA define an early, detectable oxidative dyslipidemia phenotype characterized by the convergence of lipotoxic and oxidative pathways, leading to endothelial dysfunction, nitric oxide (NO) depletion, platelet activation, and impaired placental perfusion. We further delineate druggable mechanistic nodes within this axis and outline a precision pharmacology framework for biomarker-enriched prevention trials. These nodes include cyclooxygenase-1 (COX-1)–mediated platelet activation (targeted by low-dose aspirin), NO pathway dysregulation (amenable to NO-supportive strategies), and xanthine oxidase–driven oxidative amplification (a potential target of redox-modulating agents). By explicitly linking early metabolic biomarkers to defined vascular pathways, this framework reframes first-trimester screening as a platform for mechanism-guided pharmacologic investigation rather than risk categorization alone. Before clinical integration, essential steps include independent validation across diverse populations, establishment of pregnancy-specific thresholds, demonstration of incremental predictive value beyond existing screening algorithms, and evidence that biomarker-guided strategies improve maternal and perinatal outcomes. Overall, these observations should be considered hypothesis-generating and require further validation before AIP-based assessment can support biologically informed, phenotype-guided prevention strategies within obstetric pharmacology.

## Introduction

Preeclampsia affects approximately 3%–8% of pregnancies worldwide (Cresswell et al., 2025; W.H. Organization, 2025) and remains a major contributor to maternal and perinatal morbidity and mortality, with important short- and long-term health consequences for both mother and offspring (Conley, 2024; Goffin et al., 2018; Amelia et al., 2025). Traditionally conceptualized as a disorder arising from abnormal placentation, preeclampsia is increasingly recognized as a systemic syndrome reflecting pre-existing vascular, inflammatory, and metabolic susceptibility (Kutllovci Hasani et al., 2025; Martini et al., 2025). This shift in understanding reframes preeclampsia not as an isolated obstetric complication, but as the clinical manifestation of latent biological vulnerability exposed by the physiological demands of pregnancy. In this context, pregnancy functions as a dynamic cardiometabolic stress test, capable of revealing underlying disease risk long before overt pathology becomes clinically apparent (Boyd, 2023). Despite this evolving paradigm, early pregnancy risk assessment continues to rely heavily on body mass index (BMI), obstetric history, and basic clinical parameters (Pavanya et al., 2025). While BMI provides practical population-level risk stratification, emerging evidence from metabolic medicine highlights important limitations (Santiago-Arango et al., 2025; Tanasescu et al., 2025). BMI primarily quantifies adipose tissue mass but does not assess metabolic function (Poniedzialek-Czajkowska et al., 2023). Consequently, BMI cannot distinguish between metabolically healthy and unhealthy individuals at any given weight, nor does it capture qualitative abnormalities in lipid metabolism, oxidative balance, insulin sensitivity, or endothelial function, processes central to both placental development and maternal vascular adaptation (Ashtary-Larky et al., 2023). This gap is particularly significant given that substantial proportions of women who develop preeclampsia are not obese by BMI criteria, raising the question: what alternative markers might identify metabolic vulnerability in normal-weight populations? We therefore propose a shift toward qualitative metabolic assessment that prioritizes lipid quality over body size, an increasingly recognized gap in contemporary obstetric risk stratification (Table 1).

**TABLE 1:** Comparative framework for early pregnancy risk stratification: From anthropometric Quantity to metabolic quality.

Feature	Current BMI-Centric model	Proposed oxidative dyslipidemia framework
Primary marker	BMI	AIP (log_10_ TG/HDL-C)
Secondary/conditional marker	Not routinely assessed	UA (μmol/L) as an oxidative modifier
Metabolic focus	Adipose quantity/Body size	Qualitative lipid dysfunction and oxidative stress
Detection window	Pre-conception or first prenatal visit	6–14 weeks of gestation (routine first-trimester window)
Mechanistic biological target	Obesity-associated low-grade inflammation	Endothelial vulnerability, sdLDL-mediated lipotoxicity, and reduced NO bioavailability
Clinical utility	Population-level risk categorization	Refined metabolic risk stratification and targeted surveillance
Sensitivity in non-obese women	Limited	Preserved/enhanced
Role in clinical decision-making	Broad screening	Identification of high-risk metabolic phenotypes within ostensibly low-risk groups
Intended function	Baseline anthropometric classification	Complementary, biology-informed refinement of existing risk models

Atherogenic index of plasma (AIP) is a functional proxy for triglyceride-rich lipoproteins and small dense low-density lipoprotein (sdLDL) particles and reflects lipid-driven metabolic stress rather than adiposity. Uric Acid (UA) is not proposed as a standalone screening biomarker but as a conditional modifier that refines risk stratification among women with elevated AIP. This framework is designed to complement, not replace, existing clinical risk models. BMI, body mass index; NO, nitric oxide.

Analogous challenges have long been identified in endocrine and metabolic medicine (Betlejewska et al., 2025). A growing body of preliminary data indicates that cardiometabolic disease frequently develops in individuals who do not meet traditional BMI criteria for overweight or obesity (Tanasescu et al., 2025; Ahmed et al., 2025; Dash, 2025). The concept of “metabolically unhealthy normal weight” has highlighted the inadequacy of adiposity-based metrics to capture lipid-driven and inflammatory risk (Ramoni et al., 2025; Molavizadeh et al., 2025; Carbone et al., 2025). Within this framework, biomarkers that reflect qualitative metabolic stress, rather than body size alone, have gained increasing attention. Among candidate biomarkers, the atherogenic index of plasma (AIP; log_10_[TG/HDL-C], where TG denotes triglycerides and HDL-C represents high-density lipoprotein cholesterol) was selected as the central lipid marker because it provides a biologically relevant proxy for triglyceride-rich lipoprotein burden and small dense LDL (sdLDL)-associated risk, is accessible and widely validated in cardiometabolic research, and may capture qualitative metabolic dysfunction not reflected by BMI alone (Jin et al., 2025; Wu et al., 2025; Wang et al., 2025a; Demirtola et al., 2024; Li et al., 2023; Zhao et al., 2025a; Cao et al., 2024; Norwitz and Loh, 2020; Du et al., 2025; Yang et al., 2025a). Although alternative indices such as TG/HDL-C and TyG have also been proposed, their comparative performance for preeclampsia prediction during pregnancy remains insufficiently established (Poveda et al., 2018; Bulbul and Yakistiran, 2025; Tang et al., 2025). As recently synthesized (Najera-Segura and Cabrera-Fuentes, 2025), there is an urgent need to redefine metabolic risk in non-obese adults (BMI <25 kg/m^2^) (Molavizadeh et al., 2025). Analysis of prospective cohorts suggests potential non-linearity in the lipid-disease relationship (Cao et al., 2024; Huang et al., 2024). Studies have identified possible inflection points at a TG/HDL-C ratio of 1.36 (Huang et al., 2024) and an AIP of −0.268 (Cao et al., 2024) in non-pregnant populations. Preliminary data from a recent prospective obstetric cohort suggest a potential interaction between atherogenic lipids and uric acid (UA > 198 μmol/L), with this threshold derived from first-trimester risk stratification analyses in that population (Chen et al., 2025a; Zhao et al., 2025b; Shen et al., 2025; Li et al., 2025). All proposed thresholds are provisional and derived from non-pregnant populations or limited single-cohort data and should not be applied clinically without prospective validation in pregnancy. These observations have led to the hypothesis that lipid toxicity may not progress linearly but could intensify when combined with oxidative stress. Importantly, the predictive value of AIP is not confined to lean populations (Andraschko et al., 2025). AIP has been associated with prediabetes, metabolic syndrome, and Type 2 diabetes mellitus (T2DM) across normal-weight, overweight, and obese individuals (Najera-Segura and Cabrera-Fuentes, 2025; Chen et al., 2025b; Ma et al., 2025). This consistency across the adiposity spectrum supports the interpretation of AIP as a marker of lipid-driven metabolic stress rather than a proxy for body fat mass. Such evidence has contributed to broader calls to reconsider BMI-centric definitions of metabolic risk, including a recent consensus (The Lancet Diabetes, 2025), which emphasizes metabolic dysfunction and biomarker-based assessment over anthropometric classification alone.

These advances in metabolic risk stratification are directly relevant to pregnancy. Gestation is characterized by profound physiological adaptations, including progressive insulin resistance, hyperlipidemia, and increased oxidative stress, all of which are necessary to support fetal growth but place substantial demands on maternal vascular and metabolic systems (Lourenço et al., 2025). In women with limited metabolic reserve, these adaptive processes may become maladaptive, leading to endothelial dysfunction, placental insufficiency, and hypertensive disorders of pregnancy (Dimitriadis et al., 2023; Shaw et al., 2024). Yet, the tools currently used to assess early pregnancy risk remain poorly aligned with this biological reality. Recent first-trimester mechanistic frameworks integrating angiogenic, uteroplacental, and vascular domains have improved early prediction, but they still omit metabolic and oxidative pathways central to maternal vascular adaptation (Torres-Torres et al., 2026).

Integrating insights from endocrine and metabolic research into obstetric screening frameworks offers an opportunity to refine early risk identification. Biomarkers such as AIP, which reflect qualitative lipid abnormalities and metabolic stress, may provide clinically meaningful information well before the onset of overt disease. Understanding how such markers function in early pregnancy and how they interact with established metabolic and oxidative pathways is a critical step toward more precise, biologically informed approaches to preeclampsia risk stratification. This article is a hypothesis-generating perspective and does not aim to provide a systematic or exhaustive review.

## Scope and methodological approach

This article presents a narrative perspective that poses hypotheses and does not follow a systematic review methodology. The analyzed literature was compiled through targeted searches in PubMed, Scopus, and Web of Science using the keywords: “preeclampsia,” “plasma atherogenic index,” “uric acid,” “oxidative stress,” and “pregnancy.” The literature search included publications available up to March 2026 and was restricted to English-language articles. Study selection was based on author consensus and conceptual curation, prioritizing evidence relevant to a) lipid-related metabolic risk and AIP, b) metabolic adaptations during pregnancy, and c) uric acid and oxidative stress pathways. Inclusion and exclusion criteria, as well as the PRISMA framework, were not applied because the objective was not to generate a comprehensive systematic synthesis. The evidence discussed reflects illustrative rather than exhaustive coverage and should not be interpreted as a formal assessment of consistency across all available studies.

## Population context and evidence limitations

Before synthesizing the evidence, several important contextual limitations merit acknowledgment. First, the available literature is geographically and ethnically unbalanced. Most studies linking AIP to preeclampsia derive from Asian and European populations, with limited representation from African, Latin American, and Indigenous populations. Given known ethnic variations in lipid metabolism (Raphael et al., 2024), preeclampsia phenotypes, and uric acid handling, these findings may not generalize universally. The particularly limited data from African populations highlight a critical gap and underscore the need for region-specific validation (Abdulkadir et al., 2025; Agu et al., 2024).

Second, the combined oxidative dyslipidemia construct (AIP with UA) has not been independently validated. No studies have specifically evaluated this construct across diverse populations, limiting assessment of its mechanistic and clinical generalizability. In addition, optimal AIP thresholds are likely to vary by population and gestational age, and current proposed thresholds derive primarily from non-pregnant cohorts or limited single-population studies. The proposed AIP-UA interaction currently rests on limited observational evidence from a single cohort and requires independent replication before it can inform clinical practice (Zhao et al., 2025a). These limitations do not invalidate the conceptual framework but do temper conclusions and emphasize the conditional nature of current evidence.

## AIP as an early marker of preeclampsia risk

Prospective obstetric cohort studies now provide emerging evidence that AIP, measured during the first-trimester pregnancy (≤14 weeks of gestation), is associated with the subsequent development of preeclampsia (Zhu et al., 2025). Across several prospective cohorts, emerging evidence suggests that AIP is associated with preeclampsia risk, with some studies reporting a generally linear relationship in which each standard deviation increase corresponds to a higher incidence of preeclampsia (Elovitz et al., 2025; Mitchell-Sparke et al., 2025; Mo et al., 2025). Importantly, multivariable analyses accounting for maternal age, parity, blood pressure, and BMI suggest that this association may be independent of traditional obstetric and anthropometric risk factors (Alcock et al., 2025; Cabrera-Fuentes and Boisvert, 2025; Zablah et al., 2026).

Beyond conventional regression models, machine-learning approaches further underscore the relevance of AIP for early risk stratification (Chen et al., 2025c). Random forest analyses in selected studies have ranked AIP among the more influential predictors of preeclampsia, often outperforming individual lipid parameters and anthropometric indices (Zhao et al., 2025a). This pattern aligns with recent pediatric cohort data showing that AIP independently predicts hypertension in machine-learning models, underscoring its stability as an early marker of vascular–metabolic stress (Zhu et al., 2026). Crucially, the association between first-trimester AIP and preeclampsia risk emerges well in advance of clinical disease onset (Zhao et al., 2025a; Zhao et al., 2025b; Zhang et al., 2024). In prospective cohorts, elevated AIP is detectable at 6–14 weeks of gestation, whereas clinical diagnosis of preeclampsia typically occurs in the late second or third trimester (Zhao et al., 2025b; Andresen et al., 2025; Carr et al., 2025). This temporal separation, often spanning several weeks to months, supports the concept that lipid-driven vascular vulnerability precedes overt placental dysfunction and maternal endothelial failure. From a pathophysiological perspective, early dyslipidemia may impair spiral artery remodeling, promote oxidative stress, and reduce nitric oxide (NO) bioavailability, thereby limiting the maternal cardiovascular system’s ability to adapt to the increasing hemodynamic demands of pregnancy (Kutllovci Hasani et al., 2025; Herzog et al., 2025).

Taken together, these findings support first-trimester AIP as an early and readily obtainable biomarker of preeclampsia risk. Further validation across diverse populations remains necessary.

## UA as an oxidative modifier of lipid-associated risk

UA has traditionally been regarded as a marker of disease severity in established preeclampsia, often rising in parallel with worsening hypertension, renal dysfunction, and placental insufficiency (Arias-Sanchez et al., 2025). This historical view has limited its perceived utility in early risk stratification. However, emerging prospective evidence supports a more nuanced role for UA during early pregnancy, particularly as a modifier of lipid-associated vascular risk rather than as a standalone predictor (Abu-Awwad et al., 2023; Zhao et al., 2024).

Limited first-trimester data suggest that UA may not serve as a primary independent predictor of preeclampsia (MacDonald et al., 2022; Ng et al., 2024; Alfaki Ahmed et al., 2025). Rather, preliminary evidence from a recent cohort indicates potential interaction with AIP, such that elevated AIP may confer greater risk when accompanied by higher UA concentrations (Zhao et al., 2025a). The proposed AIP-UA interaction is currently supported by limited observational evidence from a single cohort and should be considered exploratory pending independent replication. This pattern is consistent with a synergistic model in which oxidative stress modulates lipid-associated vascular risk. However, the robustness of this interaction term, its generalizability across populations, and its clinical utility relative to simpler AIP-only models all require rigorous validation.

The biological plausibility of this modifier effect is well supported. Although UA functions as an antioxidant in extracellular environments, intracellular accumulation promotes oxidative stress by activation of nicotinamide adenine dinucleotide phosphate (NADPH) oxidase, mitochondrial dysfunction, and generation of reactive oxygen species (ROS) (Ansari et al., 2025; Bortolotti et al., 2025). UA has also been shown to impair endothelial NO synthase (eNOS) activity, reducing NO bioavailability and limiting vasodilatory capacity (Park et al., 2013; Tran et al., 2022). In pregnancy, where endothelial adaptation is essential to accommodate increased blood volume and placental perfusion, these effects may critically undermine vascular resilience (Ahrens and Singer, 2025).

In this context, triglyceride-rich lipoproteins and small, dense low-density lipoproteins, as indicated by elevated AIP, may exert greater endothelial toxicity in the presence of concurrent oxidative stress. The result is a metabolic–vascular environment characterized by reduced antioxidant buffering capacity, impaired endothelial signaling, and heightened susceptibility to placental hypoperfusion. Importantly, this interaction becomes detectable early in gestation, preceding the clinical manifestations of hypertension and proteinuria by several weeks.

This pattern closely parallels observations in metabolic disease outside of pregnancy. In non-obese adults, oxidative and inflammatory states have been shown to intensify the diabetogenic and vasculotoxic effects of dyslipidemia, accelerating insulin resistance and endothelial dysfunction. In these settings, lipid indices often show stronger associations with disease outcomes when oxidative or inflammatory markers are elevated, reinforcing the notion that metabolic context modulates lipid toxicity.

Together, current evidence supports interpreting UA in early pregnancy not as a primary screening biomarker, but as an oxidative modifier that refines risk stratification among women with elevated AIP. This conditional role aligns with contemporary approaches in metabolic medicine and suggests that combined assessment of lipid burden and oxidative stress may better capture the biological processes underlying preeclampsia risk than either marker alone.

## The oxidative dyslipidemia phenotype: a unifying framework

**FIGURE 1:**
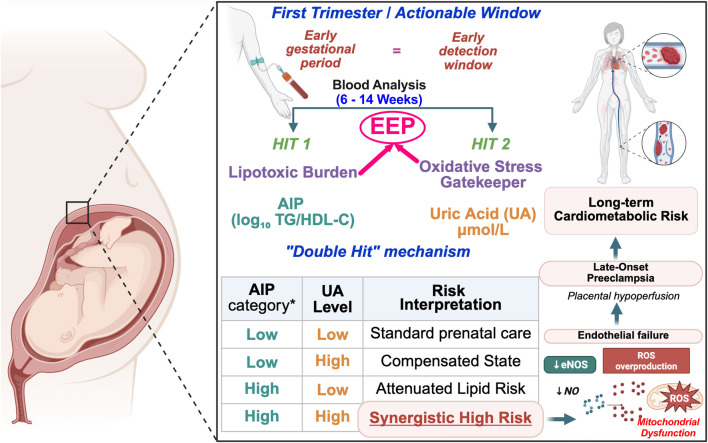
The oxidative dyslipidemia phenotype and the “double-hit” pathophysiological framework (conceptual, hypothesis-generating). This schematic illustrates a proposed, hypothesis-generating model describing the synergistic interaction between atherogenic lipid burden and oxidative stress in early pregnancy. During the first trimester (weeks 6–14; actionable window for risk stratification), the oxidative dyslipidemia phenotype may be detectable through combined assessment of the atherogenic index of plasma (AIP) and uric acid (UA). Hit one (elevated AIP) reflects lipotoxic stress characterized by an increased proportion of triglyceride-rich lipoproteins and small dense low-density lipoprotein (sdLDL) particles with enhanced endothelial penetrance (EEP). Hit two (elevated UA) functions as an oxidative modifier that may lower the threshold at which lipid abnormalities translate into vascular injury by promoting intracellular oxidative stress, mitochondrial dysfunction, and reduced endothelial nitric oxide (NO) bioavailability. The convergence of these processes is proposed to impair endothelial adaptation and placental perfusion, increasing susceptibility to placental ischemia and the later clinical manifestation of preeclampsia. Beyond pregnancy, this framework situates oxidative dyslipidemia as a potential sentinel phenotype reflecting persistent cardiometabolic vulnerability across the life course. Importantly, this figure represents a conceptual model intended to generate testable hypotheses; the existence, thresholds, and clinical utility of this phenotype require validation in independent prospective cohorts. TG, triglycerides; HDL-C, high-density lipoprotein cholesterol; eNOS, endothelial nitric oxide synthase.

We propose that the combination of elevated AIP and increased UA may define an oxidative dyslipidemia phenotype, a state characterized by atherogenic lipids in the context of heightened oxidative stress (Figure 1/Graphical Abstract). All mechanistic links described here are biologically plausible but largely inferred from associative human data and supporting experimental studies. If this phenotype exists as a discrete clinical entity with predictive value beyond AIP alone, it would suggest that oxidative stress modulates the threshold at which dyslipidemia becomes harmful. However, this remains a working hypothesis requiring validation through: (1) confirmation of statistical interaction in independent cohorts, (2) demonstration of biological plausibility through mechanistic studies, and (3) evidence of clinical utility through prospective risk prediction studies. Rather than reflecting excess adiposity, this phenotype captures a state of heightened vascular vulnerability driven by interaction between triglyceride-rich lipoproteins, reduced HDL-mediated antioxidant capacity, and oxidative stress. Increasing evidence suggests that this combined metabolic profile represents a common upstream pathway linking seemingly distinct clinical conditions, including non-obese T2DM and preeclampsia.

When accompanied by elevated UA, this risk appears to be amplified, consistent with a model in which oxidative stress lowers the threshold at which dyslipidemia becomes biologically harmful. Such interactions are well described in experimental and clinical studies, which demonstrate that oxidative stress potentiates lipid-induced endothelial dysfunction, mitochondrial injury, and inflammatory signaling. The oxidative dyslipidemia phenotype thus provides a mechanistic bridge between lipid abnormalities and vascular pathology, offering explanatory coherence across diverse disease states.

In pregnancy, this phenotype is particularly relevant. Gestation imposes substantial metabolic and hemodynamic demands, including progressive insulin resistance, physiological hyperlipidemia, and increased oxidative stress. Recent mechanistic models improve early classification but omit metabolic and oxidative pathways, emphasizing the unique contribution of the oxidative dyslipidemia phenotype (Torres-Torres et al., 2026). In women with preserved metabolic reserve, these changes are well tolerated. However, in those with pre-existing oxidative dyslipidemia, the capacity to adapt may be exceeded early in pregnancy, resulting in impaired endothelial function, abnormal placentation, and heightened susceptibility to hypertensive disorders. Importantly, this phenotype is detectable during the first-trimester, often several weeks before clinical features of preeclampsia emerge, providing a biologically meaningful window for early risk identification. Conceptually, this framework extends BMI-centered risk assessment by incorporating metabolic and oxidative features that may better characterize vulnerability in early pregnancy.

By integrating AIP and UA into a unified phenotype, this framework also facilitates continuity across the life course. Preeclampsia is a recognized sentinel event for future cardiovascular disease and diabetes, and identification of oxidative dyslipidemia during pregnancy may reflect a persistent metabolic vulnerability rather than a transient gestational phenomenon. As such, this phenotype offers a conceptual and biological link between pregnancy complications and long-term cardiometabolic health. In summary, the proposed oxidative dyslipidemia phenotype integrates lipid, oxidative, and vascular pathways into a coherent mechanistic framework, but requires independent validation.

## Implications for early pregnancy screening

This Perspective provides the first integrative framework linking the AIP and UA as a unified early pregnancy phenotype. By conceptualizing this interaction as an oxidative dyslipidemia axis, it connects lipid-driven vascular stress with redox-mediated amplification within a coherent mechanistic model. This framework further establishes a foundation for precision pharmacology by aligning early biomarkers with discrete, testable therapeutic targets and biomarker-enriched trial design.

Before any clinical integration can be recommended, several critical validation steps are required:

1. *Independent Cohort Validation:* Replication of AIP-preeclampsia associations in ethnically diverse populations with standardized outcome definitions and adequate sample sizes for subgroup analysis.
2. *Threshold Determination:* Prospective derivation and validation of pregnancy-specific, gestation-adjusted AIP cutoffs, given physiologic hyperlipidemia throughout gestation.
3. *Incremental Value Assessment:* Head-to-head comparison with existing first-trimester screening tools (PAPP-A, PlGF, sFlt-1, maternal factor algorithms) with formal assessment of sensitivity, specificity, positive predictive value, and net reclassification improvement.
4. *UA Interaction Confirmation:* Independent validation of the AIP × UA interaction effect with evaluation of whether combined assessment outperforms AIP alone.
5. *Implementation Science:* Cost-effectiveness modeling, workflow integration studies, and assessment of whether enhanced risk stratification leads to actionable clinical decisions and improved outcomes.

Taken together, these priorities indicate that any clinical role for AIP will depend on robust validation, pregnancy-specific thresholds, and demonstration of incremental value beyond existing screening models. Recent first-trimester mechanistic models integrating angiogenic, uteroplacental, and maternal vascular domains improve prediction (Torres-Torres et al., 2026), but they still omit metabolic and oxidative pathways that AIP and UA may uniquely capture. In this context, emerging evidence suggests that complementary biomarkers may help refine the identification of women in whom elevated AIP reflects true pathophysiologic vulnerability rather than physiologic gestational lipid changes. Among these, UA has gained attention as a potential modifier of lipid-associated vascular stress, providing an opportunity to move from single-marker screening to a more nuanced, phenotype-based approach.

UA measurement appears to be most informative when interpreted conditionally. Rather than functioning as a universal standalone screening marker, UA refines risk assessment among women with elevated AIP by identifying those in whom lipid-associated vascular stress is likely amplified by oxidative burden. This stratified interpretation aligns with best practices in metabolic and endocrine medicine, where biomarkers are increasingly used in combination to define risk phenotypes rather than applied indiscriminately (Table 2). Such an approach also minimizes unnecessary testing and reduces the likelihood of overclassification in low-risk populations.

**TABLE 2:** Proposed research framework for AIP-Based risk stratification - thresholds requiring validation.

AIP category*	UA level^#^	Risk interpretation	Suggested clinical response
Low	Low	Baseline metabolic risk	Standard prenatal care
Low	High	Oxidative-predominant state	Routine care with awareness of the oxidative stress context
High	Low	Lipid-predominant vulnerability	Early metabolic counseling and routine surveillance
High	High	Oxidative dyslipidemia phenotype	Eligibility for intensified monitoring and enhanced surveillance pathways

IMPORTANT: this matrix presents a research framework, NOT, validated clinical guidance. All thresholds are PROVISIONAL, and derived from non-pregnant populations or single preliminary cohorts. * AIP, threshold (−0.268): from non-pregnant metabolic cohorts; pregnancy-specific thresholds unknown. # UA, threshold (198 μmol/L): from limited preliminary data; interaction effect unconfirmed. Clinical utility, optimal thresholds, and response strategies all require prospective validation before implementation. This framework is presented to guide future research, not current clinical practice.

Importantly, the current state of evidence supports thoughtful evaluation and integration rather than immediate, universal implementation. Before routine adoption, several prerequisites should be met. These priorities include validation across ethnically and geographically diverse populations, establishment of pregnancy-specific and population-appropriate cutoff values, and clear demonstration that AIP, either alone or in combination with uric acid, confers incremental predictive value beyond existing clinical, biochemical, and angiogenic risk models. In this context, while the threshold of −0.268 serves as a robust evidence-based starting point derived from non-pregnant cohorts, the inherent physiological hyperlipidemia associated with advancing gestation suggests that future clinical guidelines may necessitate the development of gestation-adjusted scales to maintain optimal diagnostic precision. Addressing these criteria will be essential to ensure that incorporation of these markers enhances, rather than complicates, early pregnancy risk assessment and contributes meaningfully to improved maternal and perinatal outcomes.

## Pharmacological relevance of early oxidative dyslipidemia identification

From a pharmacological perspective, early identification of an oxidative dyslipidemia phenotype may have important implications for biomarker-guided prevention strategies in pregnancy, even in the absence of immediate therapeutic intervention (Xu et al., 2025). Current pharmacologic approaches to preeclampsia prevention, most notably low-dose aspirin, are applied using broad clinical risk criteria that incompletely capture underlying metabolic and vascular vulnerability (Ganem et al., 2025; George et al., 2025). Refinement of early risk stratification using lipid-based indices, such as AIP, could enable more precise identification of women in whom pharmacologic prevention is most likely to yield benefit, thereby improving risk–benefit balance and minimizing unnecessary exposure. Beyond pregnancy, detection of oxidative dyslipidemia during early gestation may also inform postpartum pharmacologic risk management, including targeted lipid-lowering or metabolic interventions in women otherwise classified as low risk by anthropometric criteria (Jiang et al., 2025; Kosmas et al., 2025). Importantly, the present evidence does not support pharmacologic intervention based on AIP or UA levels alone; rather, these biomarkers should be viewed as tools for risk enrichment that may enhance the design, targeting, and interpretability of future pharmacologic trials aimed at preventing preeclampsia and its long-term cardiometabolic sequelae.

## Broader implications beyond pregnancy and future research directions

Preeclampsia is a well-established sentinel event for future cardiovascular disease and T2DM, reflecting persistent vascular and metabolic vulnerability that extends far beyond the index pregnancy. Identification of an oxidative dyslipidemia phenotype during early gestation may therefore function as an early life-course signal of long-term cardiometabolic risk rather than a transient pregnancy-specific abnormality. Pregnancy represents a unique and currently underutilized window in which metabolic stress is physiologically amplified, allowing latent dyslipidemia and oxidative imbalance to become clinically detectable. Recognition of this phenotype at an early stage could inform not only obstetric surveillance but also post-partum risk communication, longitudinal metabolic follow-up, and targeted prevention strategies to reduce future cardiometabolic burden in women otherwise classified as low risk by traditional criteria.

Realizing this potential will require focused and coordinated research efforts. Key priorities include validation of AIP-based risk stratification across diverse ethnic and geographic populations, harmonization of pregnancy-specific AIP cut-off values, and time-to-event analyses to quantify the lead time for reliably predicting preeclampsia. Integration of AIP and uric acid with established angiogenic and clinical risk markers will be essential to define their incremental value within existing screening frameworks. Finally, interventional studies are needed to determine whether modification of AIP through lifestyle or pharmacological approaches can meaningfully alter preeclampsia risk. Collectively, such work will determine whether AIP evolves from a promising candidate biomarker into a clinically actionable component of routine prenatal and long-term cardiometabolic care.

## Pharmacologic targeting of the oxidative dyslipidemia axis: from mechanism to precision prevention

**FIGURE 2:**
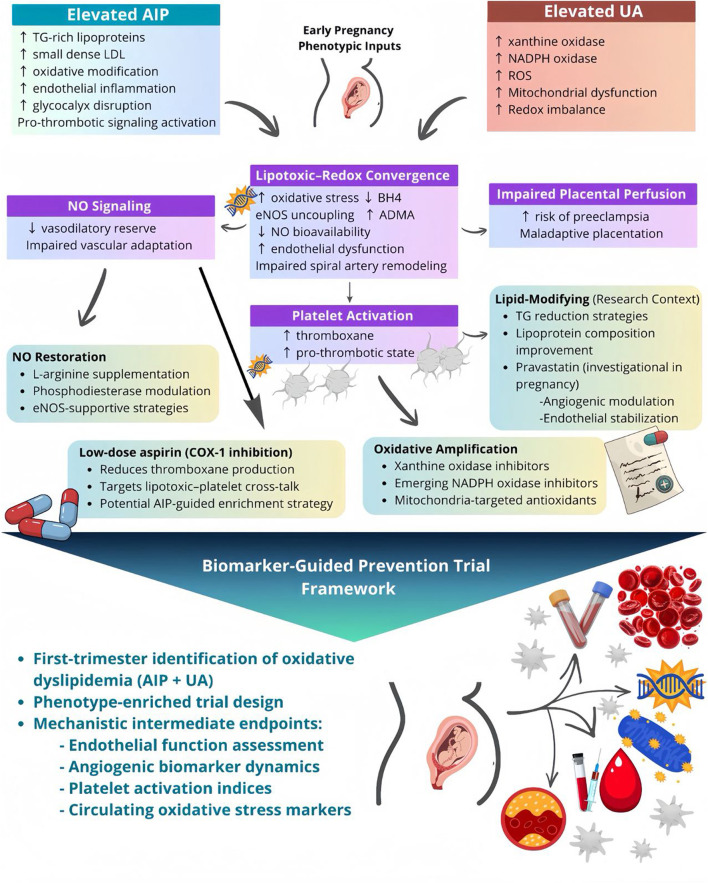
Pharmacologic modulation of the oxidative dyslipidemia axis in early pregnancy (conceptual framework). This schematic illustrates the convergence of a lipotoxic axis, reflected by elevated atherogenic index of plasma (AIP), and an oxidative axis, reflected by elevated uric acid (UA), in driving endothelial dysfunction during early gestation. Elevated triglyceride-rich lipoproteins and small dense Low-Density Lipoprotein (LDL) particles promote endothelial inflammation and oxidative modification, while uric acid activates xanthine oxidase and Nicotinamide Adenine Dinucleotide Phosphate (NADPH) oxidase pathways, amplifying intracellular reactive oxygen species (ROS). These processes converge on a lipotoxic–redox axis characterized by oxidative stress, endothelial nitric oxide synthase (eNOS) uncoupling, tetrahydrobiopterin (BH4) depletion, increased asymmetric dimethylarginine (ADMA), reduced nitric oxide (NO) bioavailability, and impaired spiral artery remodeling. The downstream vascular consequences include reduced NO signaling, diminished vasodilatory reserve, heightened platelet activation with thromboxane excess, and impaired placental perfusion, associated with maladaptive placentation and an increased risk of preeclampsia. Each mechanistic domain corresponds to pharmacologic targets that are established or investigational within pregnancy medicine, including low-dose aspirin (COX-1 inhibition), NO-supportive strategies (e.g., L-arginine or phosphodiesterase modulation), xanthine oxidase inhibition and emerging redox-modulating therapies, and lipid-modifying approaches in research settings. The lower panel outlines a biomarker-guided prevention trial framework in which first-trimester identification of oxidative dyslipidemia (AIP + UA) enables a phenotype-enriched trial design that incorporates mechanistic intermediate endpoints, such as endothelial function, angiogenic biomarkers, platelet activation indices, and circulating oxidative stress markers.

The identification of an oxidative dyslipidemia phenotype in early pregnancy (Kosmas et al., 2025; Shrestha et al., 2019) does more than refine risk stratification; it delineates a set of convergent molecular pathways that are pharmacologically addressable. By integrating lipid-driven endothelial stress (captured by AIP) with oxidative amplification (reflected by UA), this framework defines a biologically coherent axis linking triglyceride-rich lipoproteins, redox imbalance, NO depletion, platelet activation, and impaired placental vascular adaptation. Importantly, this axis corresponds to discrete therapeutic nodes that may inform biomarker-guided prevention strategies. Figure 2 illustrates the proposed convergence of lipotoxic and oxidative pathways and maps pharmacologic classes onto defined mechanistic targets. Rather than suggesting immediate therapeutic escalation, this framework provides a structured rationale for precision-enriched trial design. Elevated AIP reflects a predominance of triglyceride-rich lipoproteins and small dense low-density lipoprotein particles, which exhibit enhanced endothelial penetrance and susceptibility to oxidative modification (Dhananjaya et al., 2026). These lipoproteins promote endothelial inflammation, glycocalyx disruption, and activation of pro-thrombotic signaling pathways (Ramírez-Melo et al., 2025). Concurrently, intracellular accumulation of UA activates xanthine oxidase (XO) and NADPH oxidase systems, amplifying ROS generation and promoting mitochondrial dysfunction (Bortolotti et al., 2025; Liang et al., 2025). The resulting pro-oxidant state promotes eNOS uncoupling, diminishes NO bioavailability, and compromises vasodilatory reserve during the critical window of spiral artery remodeling (Gonzalez et al., 2025; Mandalà, 2025). These processes converge on three central vascular consequences: diminished NO signaling, heightened platelet activation, and impaired placental perfusion (Abdalla et al., 2025; Opichka et al., 2021; Torres-Torres et al., 2024). Each node within this cascade corresponds to pharmacologic targets that are either established, investigational, or conceptually plausible within pregnancy medicine.

Cyclooxygenase-1 (COX-1) inhibition with low-dose aspirin, currently the most widely adopted preventive therapy for preeclampsia, directly addresses platelet activation and thromboxane excess (Yutao et al., 2026). Irreversible blockade of platelet COX-1 reduces the synthesis of thromboxane A2 (TXA2) throughout the life of the platelet, while the endothelium preserves to a greater extent the production of prostacyclin (PGI2), favoring a shift of the TXA2/PGI2 balance towards an antiaggregatory and vasodilatory phenotype (Guthikonda et al., 2007; Maree et al., 2005; Ying et al., 2009). Within the oxidative dyslipidemia framework, women with elevated AIP may represent a subgroup characterized by amplified lipotoxic–platelet cross-talk and endothelial instability (Guo et al., 2026). If validated, AIP-guided enrichment may increase the therapeutic yield of aspirin prophylaxis by identifying women in whom platelet activation is mechanistically coupled to metabolic and oxidative stress. Such a strategy would not expand treatment indiscriminately but could refine pharmacologic targeting within existing preventive paradigms. Restoration of NO bioavailability represents a second therapeutic axis. Oxidative depletion of tetrahydrobiopterin (BH4) (Traeger et al., 2025), elevation of asymmetric dimethylarginine (ADMA) (Yang et al., 2025b), and eNOS uncoupling (Kim and Lee, 2025) are mechanistic consequences of combined lipid and UA–mediated stress. Strategies aimed at enhancing NO signaling, including L-arginine supplementation (Abdalla et al., 2025) or downstream phosphodiesterase modulation (Opichka et al., 2021; Torres-Torres et al., 2024), have been explored in limited contexts but not evaluated within phenotype-defined populations. Modulation of the Nitric oxide-cyclic 3′-5′ guanosine monophosphate (NO–cGMP) pathway with phosphodiesterase type 5 (PDE-5) inhibitors (e.g., sildenafil) was investigated as a strategy to enhance uteroplacental perfusion. However, randomized trials in severe fetal growth restriction did not demonstrate clinical benefit and identified an increased incidence of neonatal pulmonary hypertension (Pels et al., 2020; Panda et al., 2014). These findings suggest that downstream augmentation of NO signaling, in the absence of correction of upstream oxidative and metabolic drivers, may be insufficient to restore vascular adaptation. Within this context, stratification by oxidative dyslipidemia status may help clarify whether prior inconsistent results reflect underlying biological heterogeneity rather than intrinsic pharmacologic inefficacy, thereby informing a more selective and mechanistically aligned investigational approach.

A third pharmacologic node involves modulation of oxidative amplification pathways. UA-driven activation of XO contributes to ROS generation and endothelial injury (Bortolotti et al., 2025). XO inhibition with agents such as allopurinol has been investigated in selected perinatal settings (Niu et al., 2018), primarily to mitigate oxidative stress (Wang et al., 2025b). Within the proposed framework, elevated AIP combined with increased UA may identify a subgroup in whom redox-modulating strategies are biologically rational and warrant carefully designed mechanistic trials. Similarly, emerging NADPH oxidase inhibitors and mitochondria-targeted antioxidant approaches (Franczyk et al., 2026), although investigational, align directly with the pathophysiologic cascade described here.

Finally, lipid-modifying strategies merit consideration within a strictly research-oriented context. In non-pregnant populations, triglyceride reduction and improvement of lipoprotein composition correlate with enhanced endothelial function (Ramírez-Melo et al., 2025). Lipid-modifying strategies warrant consideration within a strictly research-oriented framework. Pravastatin has been evaluated in pilot and randomized studies of women at high risk for preeclampsia, with mechanistic data suggesting modulation of angiogenic balance and endothelial stabilization. Within the oxidative dyslipidemia framework, such interventions may be most biologically relevant in women exhibiting lipid-driven vascular stress early in gestation. In this context, the phenotype may serve as a rational enrichment strategy for future endothelial-targeted trials by identifying women in whom lipotoxic stress constitutes a primary upstream driver of placental dysfunction (Mészáros et al., 2023; Saben et al., 2014). At present, however, statin therapy in pregnancy remains investigational and should be confined to controlled clinical studies. This perspective does not advocate routine lipid-lowering therapy during pregnancy but rather emphasizes biologically informed stratification in interventional research.

To synthesize these mechanisms and corresponding therapeutic nodes, Table 3 summarizes the principal pathophysiologic domains linking oxidative dyslipidemia to preeclampsia and the pharmacologic targets aligned with each. This structured mapping emphasizes that the oxidative dyslipidemia phenotype is not a single biomarker construct but a multi-axis vascular vulnerability state that intersects platelet biology, NO signaling, lipid metabolism, and redox regulation.

**TABLE 3:** Mechanistic pathways linking oxidative dyslipidemia to preeclampsia and corresponding pharmacologic targets (hypothesis-generating framework).

Pathophysiologic domain	Molecular mechanism	Vascular consequence	Pharmacologic target	Representative agents	Evidence status in pregnancy
Lipotoxic stress (high AIP)	↑ TG-rich lipoproteins, ↑ sdLDL oxidation	Endothelial inflammation, glycocalyx disruption	Lipid modification	Omega-3 fatty acids; Pravastatin†	Limited; investigational
Oxidative amplification (high UA)	Xanthine oxidase activation; NADPH oxidase stimulation; mitochondrial ROS	Redox imbalance; endothelial injury	Xanthine oxidase inhibition; NOX modulation	Allopurinol; NOX inhibitors^a^	Early-stage/investigational
NO-depletion	eNOS uncoupling; BH_4_ oxidation; ADMA elevation	↓ Vasodilation; impaired spiral artery remodeling	NO pathway restoration	L-arginine; PDE-5 modulation^a^	Limited data
Platelet activation	Thromboxane–prostacyclin imbalance	Microvascular thrombosis; placental hypoperfusion	COX-1 inhibition	Low-dose aspirin	Established in high-risk women
Integrated endothelial instability	Combined lipid–ROS interaction	Impaired placentation	Multi-axis modulation	Combination strategies	Not yet tested in phenotype-guided trials

^a^ Not established for routine use in early pregnancy. † Use of statins in pregnancy remains investigational; not recommended outside clinical trials. Important: This table outlines biologically plausible pharmacologic targets within a hypothesis-generating framework. It does not constitute therapeutic recommendations. Nitric oxide, NO.

## Toward biomarker-guided prevention trials

The translational implication of this framework is not immediate therapeutic expansion but rather the design of precision informed clinical trials. Future investigations should determine whether first-trimester identification of oxidative dyslipidemia enriches for differential responses to aspirin, NO-supportive therapies, redox modulation strategies, or endothelial targeted agents. The incorporation of intermediate mechanistic endpoints such as endothelial function assessment, angiogenic biomarker dynamics, platelet activation indices, and circulating oxidative stress markers will be essential to demonstrate biological modulation before proceeding to large-scale outcome trials. By explicitly aligning biomarkers with defined pharmacologic targets, the oxidative dyslipidemia model reframes early pregnancy screening as a platform for mechanism guided prevention rather than risk categorization alone. A concrete example of this approach would involve a prospective, biomarker-enriched randomized trial enrolling women in the first trimester (≤14 weeks of gestation) stratified by AIP levels (e.g., highest quartile or above a predefined threshold such as −0.268). Eligible participants could be randomized to standard care versus an enriched prevention strategy (e.g., low-dose aspirin initiation or combined biomarker-guided management). Primary endpoints would include the incidence of preeclampsia, with secondary mechanistic endpoints such as angiogenic marker profiles (PlGF, sFlt-1), endothelial function indices, and platelet activation markers. Such a design would enable direct evaluation of whether oxidative dyslipidemia identifies a subgroup with differential therapeutic response, thereby operationalizing the transition from risk stratification to mechanism-informed intervention. For example, a randomized trial of low-dose aspirin initiated in the late first trimester could be prospectively stratified by AIP quartile. Such a design would permit evaluation of whether women in the highest AIP strata, representing greater lipotoxic–platelet cross-talk, derive differential benefit compared with those in lower quartiles. Pre-specified subgroup analysis, coupled with mechanistic endpoints (e.g., thromboxane metabolites, endothelial function indices), would allow assessment of biological effect modification rather than relying solely on overall treatment averages. This enrichment strategy exemplifies how early metabolic profiling could transition from risk categorization to mechanism-informed therapeutic targeting.

## Conclusion: a precision pharmacology roadmap for early pregnancy

Qualitative lipid dysfunction, as captured by the AIP, has emerged as a marker of cardiometabolic risk independent of body mass index in non-pregnant populations. Recent preliminary evidence suggests that first-trimester AIP may associate with preeclampsia risk, with UA potentially modifying this relationship. While these findings are promising, current evidence remains insufficient for clinical implementation. Positioning AIP and UA within a mechanistically defined oxidative dyslipidemia axis establishes a precision pharmacology framework that links early biomarkers to discrete, druggable vascular pathways including platelet COX-1 signaling, nitric oxide dysregulation, and oxidative amplification. Key Gaps Requiring Resolution: 1) Validation across diverse populations, 2) Pregnancy-specific threshold derivation, 3) Demonstration of incremental value over existing tools, 4) Evidence linking risk identification to improved outcomes, and 5) Cost-effectiveness assessment. If validated, AIP-based assessment could enhance early pregnancy risk stratification while enabling phenotype-guided prevention strategies in which lipid and oxidative profiling inform pharmacologic targeting and trial enrichment. However, establishing clinical utility will require well-designed prospective studies with appropriate comparator groups, standardized outcome definitions, and health economic analysis. This perspective proposes oxidative dyslipidemia, defined by the combined elevation of AIP and uric acid, as a unifying early phenotype linking metabolic and vascular dysfunction in preeclampsia. By integrating lipid-driven and redox-mediated pathways, this framework provides a mechanistically grounded basis for reinterpreting first-trimester risk assessment and identifying targets for pharmacologic intervention. While the current evidence remains limited and primarily observational, the model generates testable hypotheses for biomarker-guided prevention strategies. Future work should prioritize validation across diverse populations, establishment of pregnancy-specific thresholds, and demonstration that such approaches improve maternal and perinatal outcomes. Until then, this framework should be considered hypothesis-generating and not yet suitable for clinical application.

## Data Availability

The original contributions presented in the study are included in the article/supplementary material, further inquiries can be directed to the corresponding authors.
